# Economic Self‐Management in Mild Cognitive Impairment: Challenges and Considerations in Inflationary Economies

**DOI:** 10.1002/alz70857_100570

**Published:** 2025-12-24

**Authors:** Ignacio Flores, Santiago O´Neill, Guido Dorman, Julian Fernandez Boccazzi, Natalia Sierra Sanjurjo, Claudia Patricia Múnera Martínez, Maria Belen Martin Escandell

**Affiliations:** ^1^ Instituto de neurociencias Fundacion Favaloro, Buenos Aires, buenos aires, Argentina; ^2^ Neuroscience Institute, Favaloro Foundation, Buenos Aires, Buenos Aires, Argentina; ^3^ Neuroscience Institute Favaloro Foundation, Buenos Aires, Argentina, CABA, Argentina; ^4^ Neuroscience Institute Favaloro Foundation, Buenos Aires, Argentina; ^5^ Neuroscience Institute, Favaloro Foundation, Buenos Aires, Argentina; ^6^ Instituto de neurociencias Fundacion Favaloro, Buenos Aires, Buenos Aires, Argentina; ^7^ Neuroscience Institute Favaloro Foundation, Ciudad Autónoma de Buenos Aires, Argentina

## Abstract

**Background:**

Since November 5, 1881, Argentina has changed its currency five times, averaging 29 years per currency. Like many Latin American countries, poor economic policies have forced people to adapt to frequent changes in monetary design. Given that functionality scales include financial management items for patients with cognitive impairment, our objective was to determine the impact of currency changes on the functionality of patients with cognitive complaints, without dementia.

**Method:**

Prospective experimental study with patients attending cognitive stimulation workshops. We assessed the outcomes of a mathematical problem (handling Argentine currency) before and after the introduction of two new banknotes: $1,000 and $2,000 (Study 1, January 2020) and $10,000 and $20,000 (Study 2, January 2024). Statistical software R was used to compare pre‐ and post‐introduction results for both studies. A brief survey assessed proficiency in managing money amid these currency changes.

**Result:**

The study included patients diagnosed with Mild Cognitive Impairment (*n* = 38), averaging 71 years of age with a mean MMSE score of 28. The mathematical problem was correctly solved by 21 patients in Study 1 compared to 12, and in Study 2, 15 compared to 9. We used the non‐parametric chi‐square test to compare the problem resolution in these studies (Study 1: X^2^ = 9.34, *p* =  0.002; Study 2: X^2^ = 7.5, *p* =  0.006). Average calculation times were 13.74 seconds (Study 1, Phase 1) vs. 23.89 seconds (Phase 2), and 13.8 seconds vs. 24 seconds in Study 2. The paired Student's t‐test revealed significant differences in response times (Study 1: t = ‐6.54, *p* < 0.000001; Study 2: t = ‐4.67, *p* =  0.00036). Survey results showed that while all patients managed cash, only 10 of 38 could correctly identify new denominations (26%), and none could accurately determine the figures on each bill. Additionally, 11 patients (29%) reported difficulties managing banknotes, while only 6 (16%) did not confuse denominations.

**Conclusion:**

Currency changes and new banknote designs may affect management for patients with cognitive complaints and Mild Cognitive Impairment (without dementia). Items related to economic self‐management in functionality scales may lack specificity in countries with high inflation and constant currency deregulation.

